# Evaluation of the Effectiveness and Implementation of an Adapted Evidence-Based Mammography Intervention for African American Women

**DOI:** 10.1155/2015/240240

**Published:** 2015-10-04

**Authors:** Linda Highfield, Marieke A. Hartman, L. Kay Bartholomew, Philomene Balihe, Valerie A. Ausborn

**Affiliations:** ^1^Department of Management, Policy and Community Health, School of Public Health, University of Texas, 1200 Pressler Street, Houston, TX 77030, USA; ^2^Department of Health Promotion and Behavioral Sciences, School of Public Health, University of Texas, 7000 Fannin Street, Houston, TX 77030, USA; ^3^Episcopal Health Foundation, 500 Fannin Street, Suite 300, Houston, TX 77002, USA

## Abstract

Breast cancer mortality disparities continue, particularly for uninsured and minority women. A number of effective evidence-based interventions (EBIs) exist for addressing barriers to mammography screening; however, their uptake and use in community has been limited. Few cancer-specific studies have evaluated adapted EBIs in new contexts, and fewer still have considered implementation. This study sought to (1) evaluate the effectiveness of an adapted mammography EBI in improving appointment keeping in African American women and (2) describe processes of implementation in a new practice setting. We used the type 1 hybrid design to test effectiveness and implementation using a quasi-experimental design. Logistic regression and intent-to-treat analysis were used to evaluate mammography appointment attendance. The no-show rate was 44% (comparison) versus 19% (intervention). The adjusted odds of a woman in the intervention group attending her appointment were 3.88 (*p* < 0.001). The adjusted odds of a woman attending her appointment in the intent-to-treat analysis were 2.31 (*p* < 0.05). Adapted EBI effectiveness was 3.88 (adjusted OR) versus 2.10 (OR) for the original program, indicating enhanced program effect. A number of implementation barriers and facilitators were identified. Our findings support previous studies noting that sequentially measuring EBI efficacy and effectiveness, followed by implementation, may be missing important contextual information.

## 1. Background

Breast cancer is the most common cancer in the United States and is the second leading cause of cancer mortality in women [1, 2], with lower incidence in African American women but higher stage at diagnosis and greater mortality as compared to non-Hispanic white women [2, 3]. Enhancing guideline adherent mammography routines among these women may be important to address this disparity [3]. While a number of effective evidence-based interventions (EBIs) exist for addressing barriers to mammography screening, like other EBIs, their uptake and use in community settings have been limited [4–7]. Reasons for lack of uptake include cancer planners' anticipation of a misfit between interventions tested in controlled efficacy trials and the needs of their settings [8–10]. Both the perception of lack of fit and the possibility of real deficits in an EBI fit for a new community can be addressed by judicious and systematic adaptation of EBIs by research-practice partnerships and consultation with the community to improve fit [8–15].

Planners face the challenge of striking a balance between program fidelity, that is, implementation of an EBI as intended, and adaption to the needs of the adopting site [15]. Some efforts to promote use of evidence-based programs suggest that the primary concern should be fidelity rather than adaptation because of the lack of data to suggest that adaptation improves program effectiveness [16]. However, in a review of over 500 studies that demonstrated that program implementation affected outcomes of prevention programs, Durlak and DuPre point out that while higher levels of fidelity were closely tied to improved program outcomes, levels of fidelity were well below 100% across interventions [17]. Therefore some adaptation occurred and might have been seen as necessary for program implementation. Elliott and Mihalic have outlined four ways that programs are typically adapted: adding or deleting program components; changing program components or content; changing the process or intensity of implementation; and making cultural modifications [16]. Barrera Jr. and colleagues found that behavioral interventions were more effective when adapted for a new cultural group than usual care and other control conditions and that most planners agreed that adaptation begins with data collection, to inform the need for adaptation, and ends with testing in the new setting [18].

Best practice is to always evaluate an EBI used in a new setting, however, particularly one that has been adapted. Evaluation of adapted EBIs is recommended, since adaptation may harm the effective elements of an EBI (i.e., core elements) [11]. Besides this need for impact evaluation, there is a need to evaluate the feasibility and fidelity of intervention implementation in the new population and setting [10]. However, few cancer-specific studies have evaluated effectiveness of adapted evidence-based interventions in new contexts, and fewer still have evaluated implementation in real-world contexts specifically [19, 20]. Of the few studies that have evaluated implementation of cancer-specific EBIs, facilitators for implementation and fidelity included the use and enthusiasm of program champions, academic detailing, and training (a higher degree of control) and team involvement/communication. Barriers included lack of attendance at training sessions, incomplete exposure to EBI tools/components, and competing demands at the practice level [20]. The authors could find no published studies that discussed real-world implementation of mammography EBIs in particular.

Therefore, the objectives of this study were to (1) evaluate the effectiveness of an adapted mammography EBI in improving appointment keeping for mammography in African American women and (2) describe processes of implementation of the EBI in a practice setting. Study results will test the hypothesis in which the effectiveness of the original EBI will be retained after adaptation and provide lessons learned for future intervention implementation in the real-world setting of mammography screening.

## 2. Methods

### 2.1. Evidence-Based Intervention

For this study, we adapted the intervention “Breast Cancer Screening among Nonadherent Women,” originally developed by Duke University and Kaiser Foundation Health Plan [21]. The intervention is a tailored telephone counseling reminder based on the Transtheoretical Model of Change [22]. The program assessed a woman's stage of readiness to attend her appointment through a series of survey questions and counseled her through barriers to attendance. Following the Transtheoretical Model, the five stages were as follows: precontemplation, no intention to attend appointment; contemplation, intends to attend appointment; preparation, intends to attend appointment and is making preparations for taking action; action, has attended the appointment; maintenance, keeps attending appointments [22]. In the original trial, women who were off schedule with screening were more than twice as likely to get a mammogram if they received the telephone counseling (OR = 2.10).

We adapted the intervention using Int Map Adapt, a modified version of intervention mapping (for full details of the intervention adaptation, please see Highfield et al. also in this issue) in the following ways: (1) performing needs assessment among local African American women to identify salient barriers and include them in the barrier scripts; (2) developing a foundational communication process based on active listening to make it easier for the patient navigator to hold a real world rather than research conversation (when not dealing with a specific barrier) and to develop rapport with the patient; (3) changing assessment of stage of readiness to include only two categories precontemplation/contemplation or preparation/action and then matching the script to whether the women intended to keep her appointment or is unsure; (4) pretesting the changes with local women to assess acceptability and fine-tune scripts; and (5) developing an implementation protocol and training the navigator [14]. The adapted intervention aimed to increase scheduled mobile mammography screening appointment attendance rates among low-income African American women with care provided by a mobile mammography provider which was the largest nonprofit breast cancer screening organization in the greater Houston area. The systematic and collaborative adaptation process of the original EBI for use in local practice is reported elsewhere (see Highfield et al., this issue).

### 2.2. Study Design

We used the type 1 hybrid design to test the intervention's effectiveness and to gather information on the implementation [23, 24]. This type of design focuses on effectiveness evaluation and answers questions such as “what are possible facilitators and barriers to real-world implementation of an EBI?” and “what potential modifications could be made to maximize implementation?” in addition. We originally planned a randomized controlled trial but found that the navigator could not alternate between usual care and the adapted intervention. Therefore, we changed to a quasi-experimental, sequential recruitment design in which we assigned contacted women to usual care or adapted intervention in sequential groups of 50 patients. See enrollment and study limitations for further detail. The time period for enrollment and collection of patient data was predetermined based on funding and availability of the clinical partner and took place from February to December 2012. We sought to contact as many patients as possible within this time window. This study operated under Institutional Review Board approval from St. Luke's Episcopal Hospital Institutional Review Board.

### 2.3. Study Setting

A local mobile mammography partner served as the site for implementation of the intervention (including recruitment and data collection). In 2011, the organization provided 33,784 screening and diagnostic procedures for those able to pay; 19,369 screening and diagnostic procedures at no charge to low-income, uninsured women; and 8,857 free patient navigation services to patients without insurance. Mobile screening mammography services are provided to over 7,000 women a year, covering a 15-county region centered on Harris County, TX. Services are provided in a variety of settings, including schools, work-sites, federally qualified health centers, churches, and other community settings. The mobile mammography provider in this study serves a diverse population including Caucasians, Hispanics, Asians, African Americans, and immigrant populations. Approximately 20% of the low-income, uninsured patient population at the time of study was African American (2,200 women). The baseline expected no-show rate for uninsured, low-income African American women was 38% (unpublished data).

### 2.4. Patient Enrollment

Inclusion criteria were as follows: African American, female, age 35–64, uninsured, income of ≤200% of the federal poverty level (FPL), and an upcoming appointment for a mobile screening mammogram at a program partner site. We identified eligible patients from the electronic patient scheduling records. The patient navigator made three calls to reach all eligible patients including calls at different times of the day and weekends for those who were not reached in the initial attempt. Reached individual patients received one phone call from the patient navigator in order to deliver the intervention. We expected intervention calls would take on average 6–10 minutes. Reached individual patients were initially enrolled into each group by randomization (using a randomized controlled trial (RCT) design) from February to April 2012; however, we ran into implementation issues with the patient navigator (see Section 3), so we adjusted to a sequential enrollment procedure from May to December 2012. The navigator called patients in the comparison group and provided them with a standard appointment reminder which included the date, time, and location of their upcoming appointment. If a patient did not answer the phone, the navigator left a voicemail message containing the reminder. The navigator read to patients in the intervention group an oral consent over the phone and after consent asked the following staging question: “How confident are you that you will keep your upcoming appointment?” The navigator then counseled as needed for any barriers uncovered in the phone call per the intervention protocol. No blinding was used in this study.

### 2.5. Measures and Data Tracking

The primary outcome of appointment keeping was ascertained from mobile mammography clinical records (nonattendance = 0, attendance = 1). In addition, we collected information about sites, age of patient, number of days between reminder call and appointment, and study stage (i.e., design coded as 0/1 for randomized controlled trial versus quasi-experimental one). Appointments were scheduled to 41 different sites across 8 counties. The sites were divided into 2 categories, community sites (local nonprofit organizations or government agencies, community initiatives, schools, health fairs, or other community organizations) and hospital/clinic sites (local hospital, federally qualified health center, or charity clinic). Age and days to appointment were categorized in the following categories: 35–39, 40–49, and 50–64 years old and 0 days, 1 day, 2 days, 3-4 days, and 5 days or more between phone call and appointment.

We evaluated the secondary outcome of implementation fidelity by monitoring of intervention phone calls and comparing them to the protocol, making site visits to the mammography site, and meeting with implementation staff (researchers and practitioners). A series of three phone calls made by the patient navigator were recorded at the beginning of implementation. These recordings were evaluated by the research team for compliance in asking the staging question and using active listening and scripted responses to patient identified barriers during the phone call. Following review, the navigator received feedback on the staging question and active listening. During implementation, phone calls were periodically monitored on-site by a member of the research team for the same compliance issues. In addition, we made postintervention follow-up phone calls to a randomly selected subset of intervention patients (*n* = 50) to assess their perception of the EBI calls and systems barriers encountered (see Topic-List).

*Topic-List for Follow-Up Calls and Implementation Evaluation*

1. Is there anything you want to tell me about your mammogram appointment so we can make the experience better?
2. Do you remember talking to anyone from [mobile mammography program name] before your mammogram appointment? Do you remember who?
3. What was the conversation about?
4. What stands out about your experience talking to (navigator name)?
5. Please tell me what caused you to keep this mammogram appointment? (Probe: was there anything else?)
6. Do you remember anything specific about the conversation with (navigator name) that helped you keep your appointment? If yes, what about the conversation helped you keep your appointment?
7. Are there any other reasons you kept your mammogram appointment?
8. On a scale from 1 to 5, with 5 being most helpful, how helpful did you find your phone call with (insert navigator name)?
9. Can you think of things that would have been helpful to hear from (navigator name) that would have made the phone call better?
10. Was there anything more we could have done to help you keep your appointment?
11. Do you have any other comments regarding our study of how to get women to keep their mammogram appointments?

We tracked all data for the pilot either in an Access database or in paper data collection forms. The database included fields for a unique identifier for each patient, date and time of attempted call(s) with outcome of each (reached, not reached, left message, and bad number), barriers, and systems barriers encountered during the session, such as the patient was not aware they needed a doctor's order to receive a mammogram. We also included an open text field for the patient navigator to record notes during the call. Data available from the mammography partner's data system included age, sponsored status (lack of insurance and ≤200% FPL), site of screening, date and time of appointment, and contact information including phone number. The research assistant cleaned the data by comparing the Access database with the paper forms and existing records from the mammography partner. Any inconsistencies between the database and paper forms were investigated with the site and patient navigator for clarification. Data from both databases were combined into one Access database and exported to Stata for analysis.

### 2.6. Data Analysis

We used Stata (Stata Corp., College Station, TX, USA) for statistical analysis. We calculated descriptive statistics and then conducted logistic regression analysis to report attendance in the intervention group as compared to the comparison group. Chi-square tests (and Fisher's exact tests when cell sizes were less than five) were used to evaluate group differences between potential confounding variables, including age, days between reminder calls, mammography site (community versus clinical setting) and appointment time, and the study stage (i.e., design change). Both unadjusted and adjusted logistic regression models were fitted to determine intervention's effectiveness in improving mammography attendance. Factors in the adjusted model included, besides group (intervention; control), mammography site, age of patient, number of days between reminder call and appointment, and navigator making the reminder calls. Study stage (design) was not included in the model as it was highly collinear with navigator as only one navigator made calls during each phase. Following the basic analysis, we further evaluated the effectiveness of the EBI using intent-to-treat analysis [25–28]. In this study, we used intent-to-treat analysis which considered the outcomes (appointment attendance) for all women based on their group designation at the time of phone call attempt (intervention or control) and not just those who were reached and treated following protocol by the patient navigator. Intent-to-treat analysis ignores deviations in protocol, noncompliance, and anything that may happen after group assignment [25–28]. We conducted power analysis using a two-tailed two-sample frequencies Fisher's exact test with *α* = 0.05 and adjusted for unequal sample sizes to evaluate ability to detect a difference between the groups.

## 3. Results

**Figure 1:**
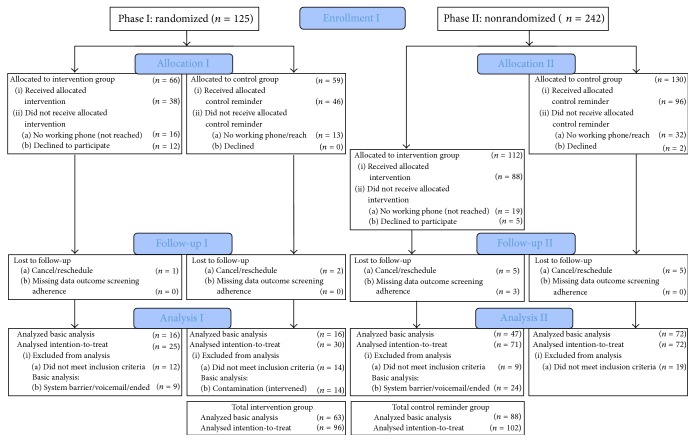
Flow chart of patient enrolment, follow-up, and basic and intention-to-treat analyses.

Figure 1 shows the CONSORT/TREND diagram with the total number of enrolled patients per study stage (randomization and sequential enrolment stage), those assigned, allocated, exposed to the intervention, followed-up, and analyzed, both in the basic effectiveness (*n* = 151) and intent-to-treat analysis (*n* = 198). The intervention and comparison groups were similar with regard to age and number of days between reminder call and appointment as shown in Table 1. The average and median age for patients in both groups was 51 years (range: 36–64). The average and median number of days between reminder call and patient appointment was 3 days for both groups (range: 0–13 days). No effect was observed for the study stage (design change) (*χ* ^2^ = 0.292). The groups were different in regard to the type of mammography site, with women in the intervention group being screened in community settings more frequently than the control group in both the basic analysis and intent-to-treat analysis (see Table 1). The no-show rate for patients in the comparison group was 44%. The no-show rate for patients in the intervention group was 19% meaning that the EBI in this study led to a 57% reduction in the no-show rate in the basic analysis (calculated as percent change).

### 3.1. Effectiveness Results

The unadjusted and adjusted results are presented in Table 2. The unadjusted odds of a woman in the intervention group attending her appointment was 3.38 times higher than for a woman in the control group (*p* < 0.001) in the basic analysis. The adjusted odds of a woman in the intervention group attending her appointment were 3.88 as compared to the control group (*p* < 0.001). No effect was found for the change in study design. In the intent-to-treat analysis, the unadjusted odds of a woman attending her appointment if she was in the intervention group were 1.84 (*p* < 0.05). The adjusted odds of a woman attending her appointment in the intent-to-treat analysis were 2.31 as compared with the control group (*p* < 0.05). With the no-show rate of 44% observed in the comparison group, using a two-tailed test and *α* = 0.05, there was 87% power to detect a change in the no-show rate to 19% in the intervention group for this study in the basic analysis.

### 3.2. Implementation Results

We encountered a number of systems barriers to implementation. These included the following: confusion about responsibility for implementation of usual care reminder calls; lack of clear communication about the prerequisites of a doctor's order and clinical exam prior to screening; and inconsistent notification about costs associated with screening.

Fourteen out of 96 (15%) patients in the intervention group reported encountering systems barriers, including the fact that they were unaware of their upcoming appointments, unaware of the need for a doctor's order to obtain a mammogram, and unaware of the out-of-pocket cost of the mammogram. Additionally, some sites reported issues with the mobile units going to the wrong location, sites being cancelled with short notice due to mechanical issues (mobile unit broke down or mammography machine needed service), and unclear communication about scheduling procedures, such as how many patients could be seen and at what time for scheduled mobile screening dates.

Of the 50 randomly selected intervention patients for follow-up phone calls to assess patient perception of the EBI 42 completed the interview (84%). In these calls, we found that 34 patients remembered their reminder phone call from the navigator (81%). The patients who remembered their call and conversation reported positive interactions with her. They reported, for example, “*She was warm, friendly, helpful, sweet, supportive and sincere*.” When asked if there was something about the phone call from the navigator that helped them to keep their appointment, 18 patients reported positive impact, such as “*The encouragement from her [the navigator] went beyond a reminder call*,” “*she cared*,” “*put me first*,” “*helped me overcome my misconceptions*,” and “*was nice*.” When asked how helpful they found the phone call, all patients that remembered the conversation (*n* = 34) rated it as 5 out of 5, except one patient who rated it 4 out of 5. Finally, the patients who attended their appointment were asked to share their thoughts of the reminder phone call program. Patients reported that “*It's important. Catch it (breast cancer) early to have a chance*,” “*They (women) need to go and have it done!*,” “*Taking care of yourself is a major point to bring up*,” “*It's a wonderful program*,” “*It's a must*,” and “*I think it's great that we are talking to women to let them know that mammograms are important*,” and recommended “*Use media and marketing to reach women without insurance*,” “*Transportation is a very big deal and would be a help. Maybe find a church with a van that could help out*,” “*Spread the news about breast health–put it in churches and schools. I was telling people at my church about the mammography program and they had never heard about it*.”

Seven of the 42 patients we conducted follow-up calls with did not attend their appointment. When asked if there was anything we could have done to have helped them keep their appointment, three reported they were sick on their appointment day, three had last minute transportation issues, and one reported that she did not have the money for the copay. Due to the nature of the mobile program, in many cases our patient navigator was not able to reschedule patients directly if during the intervention call they indicated a desire to change their appointment. Patients had to be routed through the mobile program coordinators or the site coordinators in the community in order to reschedule. This meant a loss of continuity with the patient and in some cases patients reported having difficulty reaching the coordinators to reschedule.

## 4. Discussion

This study used a hybrid type 1 design to evaluate both effectiveness of an adapted EBI in a practice setting and the implementation process. The effectiveness of the adapted EBI was 3.88 (adjusted OR) versus 2.10 (OR) for the original program [21]. This is consistent with the findings of Barrera Jr. et al., which systematically adapted EBIs improvement program effectiveness when compared to a control reminder [18]. The adapted EBI in this study reduced appointment no-shows by 57 percent from baseline in the clinical practice and would be suitable for scale-up.

Few published studies provide a detailed description of EBI effectiveness with implementation outcomes in a single study, particularly for cancer-specific EBIs [19, 20, 23]. The “Communicating Health Options Through Information and Cancer Education” (CHOICE), “Improving Systems for CRC Screening at Harvard Vanguard Medical Associates” (HVMA), and “Improving CRC Screening and Follow-up in the Veterans Health Administration” (VHA) programs all considered implementation context during their evaluations [20]. These studies encountered some of the same implementation barriers and facilitators we found in this study [20]. We found that monitoring of implementation was valuable and that the study approach needed flexibility to deal with evolving implementation issues, such as the lack of consistent standard care reminder calls and the navigator struggling with the simultaneous implementation of the control and intervention process. This was consistent with the recommendations from the Cool Pool trial where they noted that continuous monitoring of implementation was critical. Other authors have also noted the importance of continuous monitoring of implementation [20]. Additionally, the ability to identify and measure all implementation issues at the beginning of a study is limited and has been noted as a barrier in previous studies [20]. For example, in this study, we did not know prior to implementation monitoring whether reminder calls were implemented consistently. Implementation issues like these are likely to arise only once monitoring begins and may also appear over time, requiring subsequent intervention or changes in protocol to address them.

Our findings further highlight themes from previous studies which have noted that the predominant research paradigm of sequentially measuring EBI efficacy and effectiveness, followed by implementation studies, may be missing important contextual information [7, 23, 29]. We were able to find and correct problems with implementation based on the results of our process evaluation which we monitored continuously throughout the study. The process evaluation also allowed us to find problems with fidelity early in the study and correct them. The major correction was to change the design so that the navigator did not have to conduct two different interventions in the same period of time. We also added plans for an intent-to-treat analysis to increase our confidence in the validity of our results. After an unsuccessful attempt to train the original navigator to adhere to protocol, we replaced the navigator and conducted repeated trainings and monitoring of calls more frequently with the new navigator. This monitoring process may have contributed to the increased effectiveness of the EBI that we observed in this study in addition to the systematic adaptations made to the EBI.

### 4.1. Strengths and Weaknesses of the Study

This study has a number of strengths and limitations which should be considered. This study is one of only a very few studies to evaluate both EBI effectiveness and implementation in a community context and provides critical insights for the future translation of EBIs, particularly for mammography interventions. A major strength of this study was using a systematic process for adapting an EBI [11]. The process included working in a research-community partnership with an advisory board comprised of researchers and practitioners who worked together to perform a community needs assessment, select an EBI, adapt the EBI based on the needs assessment, pretest it, implement it, and evaluate it [13].

A number of limitations must also be considered for this practice-based evaluation study. First, this study was conducted in a mobile mammography practice and the findings may not be generalizable to other implementation contexts. Best practice indicates that anytime an intervention is being considered for a new population or context, that needs assessment and evaluation is needed (see Introduction and Highfield et al., 2014 [13]). Our findings show that by retaining core elements of an intervention, such as stage-based telephone counseling on barriers for mammography appointments that effectiveness can be maintained or even improved in a new population. We have no reason to believe this would not be the case when extending our intervention into a broader population context. For instance, many of the barriers to mammography screening we found among African American women are complementary to barriers to screening faced by all underserved women [30]. The most significant weakness of this study was our initial inability to train the navigator to keep the control and adapted intervention groups separate in the first study design. However, the process evaluation in this study enabled us to correct the behavior of the navigator and redesign the study from a RCT to a quasi-experimental design that we expected would be more feasible in practice. In the quasi-experimental design, we enrolled 47 patients in the intervention. The main threats to validity from this type of enrollment were selection bias, where patients reached during the sequential enrollment time period may not have been representative of the larger patient population in the clinic. We dealt with this validity threat by comparing patient demographics between the randomized and sequential design process, by including a design change variable in the regression analysis and by conducting an intent-to-treat analysis, which is useful for dealing with deviations in protocol. Also, even though this study lacked a true nonintervened group, the baseline no-show rate of 38% serves as a proxy control group since reminder calls were made only rarely. Additionally, the effect of using a control group receiving standard reminder calls as opposed to a true no-contact control group could have downward biased the results of the study. In other words, this may have made it harder to find an intervention effect in our study. Our control and intervention groups in both the basic analysis and intent-to-treat analysis differed in where they received their screening, with women in the intervention group being more likely to be screened in a community as opposed to a clinical setting. However, all women in the study were required to have a doctor's order to obtain screening and the differences were consistent in both the basic analysis and intent-to-treat analysis, so we believe the effect would be minimal on the results. Further, no significant difference was observed in the regression model between screening sites when controlling for other factors. Finally, we evaluated the EBI using patient data available from the clinical provider in this study. There may have been important factors such as educational level and occupation which we were not able to evaluate due to a lack of data availability from the provider. While these factors may be important, it is important to note that these are nonmodifiable factors and have been shown to have limited value when designing and evaluating EBI programs [30]. Lastly, women enrolled in our study were low-income and uninsured, two factors that generally correlate with education and occupation, so while we did not measure those directly, we believe their effect would have been minimal on the outcomes.

### 4.2. Lessons Learned

Interventions are rarely implemented with complete fidelity and in this study the navigator struggled to implement either intervention protocol, but especially the usual care group with fidelity. The navigator stated that she wanted to help all women attend their appointment and seemed not to be able to adhere to protocol. Even when we hired a second navigator, protocol adherence continued to be somewhat difficult. We believe the important learning from this is about staffing in a research study versus staffing in a clinical or other professional setting. In the original evaluation of an EBI in a research setting, research assistants (usually students) do not have a particularly strong professional identity or habitual way of doing tasks closely approximating the research protocol. In contrast, in a practice implementation, new protocol driven tasks are given to professional care providers who may be unable to divert from their normal practice. It is important for future researchers to consider issues of fidelity when adapting, training, monitoring, and measuring EBIs in community contexts.

Additionally, best practice indicates the need for EBI testing in new contexts (e.g., effectiveness testing in the new setting) [11, 31]; however measuring and addressing context specific implementation issues remain challenging [32–34]. Currently, there is a lack of standardized and validated measures that can be used to assess implementation [35]. Additionally, studies have noted the need for multilevel interventions that consider implementation context; however there currently is no packaged approach to implementation available in the published literature [36]. Future studies should consider creating a packaged implementation intervention which could be tested and evaluated in the context of EBI implementation in the community.

## 5. Conclusion

This study provides an example of the real-world implementation of an adapted EBI. It demonstrates best practice for adaptation and evaluation of an EBI using a hybrid type 1 design and can be used for a model of blending research and practice to increase the uptake of EBIs and to make sure that they show effectiveness in new settings.

## Figures and Tables

**Table 1:** Descriptive statistics for patients in the control versus intervention groups.

Patient characteristics	Basic analysis		Intent-to-treat analysis	
	Intervention group *n* (%)	Usual care group *n* (%)	Intervention group *n* (%)	Usual care group *n* (%)
Age group				
35–39	5 (8%)	4 (5%)	8 (8%)	5 (5%)
40–49	25 (40%)	38 (43%)	38 (40%)	41 (41%)
50–64	33 (52%)	46 (52%)	49 (52%)	53 (54%)
	*χ* ^2^ = 0.8162, *p* = 0.691^∧^		*χ* ^2^ = 0.8810, *p* = 0.670^∧^	

Mobile site				
Clinic	36 (60%)	71 (81%)	49 (57%)	73 (74%)
Community	24 (40%)	17 (19%)	37 (43%)	26 (26%)
	*χ* ^2^ = 7.6191, *p* = 0.006^*∗*^		*χ* ^2^ = 5.769, *p* = 0.016^*∗*^	

Days from call to appointment				
0	4 (5%)	4 (4%)	8 (8%)	4 (4%)
1	25 (29%)	28 (26%)	36 (38%)	29 (28%)
2	12 (14%)	20 (19%)	18 (19%)	21 (21%)
3-4	33 (39%)	44 (41%)	15 (16%)	25 (25%)
≥5	11 (19%)	12 (11%)	19 (20%)	23 (23%)
	*χ* ^2^ = 2.9846, *p* = 0.558^∧^		*χ* ^2^ = 4.948, *p* = 0.298^∧^	

Screening outcome				
Attendance	51 (81%)	49 (56%)	70 (73%)	60 (59%)
Nonattendance	12 (19%)	39 (44%)	26 (27%)	41 (41%)
	*χ* ^2^ = 10.2484, *p* = 0.001^*∗∗*^		*χ* ^2^ = 4.003, *p* = 0.045^*∗*^	

^*∗*^Statistically significant at *p* < 0.05.

^*∗∗*^Statistically significant at *p* = 0.001.

^∧^Fisher's exact test used for *p* value.

**Table 2:** Unadjusted and adjusted logistic regression results for mammography appointment attendance.

	Crude OR (95% CI)	Adjusted OR (95% CI)
Basic analysis		
Group (control versus intervention)	3.38^*∗∗*^ (1.59–7.21)	3.88^*∗∗*^ (1.70–8.86)
Age	0.997 (0.95–1.04)	0.903 (0.492–1.66)
Days between call and appointment	1.001 (0.753–1.33)	1.11 (0.799–1.54)
Navigator	1.33 (0.875–2.01)	1.25 (0.773–2.04)
Mobile site	0.843 (0.397–1.79)	0.562 (0.241–1.31)
Intent-to-treat analysis		
Group (control versus intervention)	1.84^*∗*^ (1.01–3.35)	2.31^*∗*^ (1.09–4.93)
Age	1.04 (0.642–1.68)	1.07 (0.637–1.78)
Days between call and appointment	1.03 (0.805–1.31)	1.11 (0.856–1.43)
Navigator	1.27 (0.872–1.84)	1.07 (0.684–1.67)
Mobile site	1.24 (0.665–2.33)	1.64 (0.831–3.25)

^*∗*^ *p* < 0.05.

^*∗∗*^ *p* < 0.001.
